# Antimicrobial Resistance of *Acetobacter* and *Komagataeibacter* Species Originating from Vinegars

**DOI:** 10.3390/ijerph19010463

**Published:** 2022-01-01

**Authors:** Eva Cepec, Janja Trček

**Affiliations:** 1 Department of Biology, Faculty of Natural Sciences and Mathematics, University of Maribor, SI-2000 Maribor, Slovenia; eva.cepec1@um.si; 2 Faculty of Chemistry and Chemical Engineering, University of Maribor, SI-2000 Maribor, Slovenia

**Keywords:** acetic acid bacteria, *Acetobacter*, *Komagataeibacter*, antimicrobial resistance, trimethoprim resistance, erythromycin resistance, ciprofloxacin resistance, chloramphenicol resistance, ampicillin resistance, gentamicin resistance

## Abstract

Consumers’ preference towards healthy and novel foods dictates the production of organic unfiltered bottled vinegar that still contains acetic acid bacteria. After ingesting vinegar, the bacteria come into close contact with the human microbiota, creating the possibility of horizontal gene transfer, including genetic determinants for antibiotic resistance. Due to the global spread of antimicrobial resistance (AMR), we analyzed the AMR of *Acetobacter* and *Komagataeibacter* species originating mainly from vinegars. Six antibiotics from different structural groups and mechanisms of action were selected for testing. The AMR was assessed with the disk diffusion method using various growth media. Although the number of resistant strains differed among the growth media, 97.4%, 74.4%, 56.4%, and 33.3% of strains were resistant to trimethoprim, erythromycin, ciprofloxacin, and chloramphenicol, respectively, on all three media. Moreover, 17.9% and 53.8% of all strains were resistant to four and three antibiotics of different antimicrobial classes, respectively. We then looked for antimicrobial resistance genes in the genome sequences of the reference strains. The most common genetic determinant potentially involved in AMR encodes an efflux pump. Since these genes pass through the gastrointestinal tract and may be transferred to human microbiota, further experiments are needed to analyze the probability of this scenario in more detail.

## 1. Introduction

Many genetic determinants for antimicrobial resistance (AMR) are widely spread among clinically relevant bacteria, mainly because of humans’ excessive or improper use of antibiotics and the very efficient gene exchange and adaptation mechanisms in bacteria [1]. The AMR genetic determinants are also circulating among bacteria in the natural environment and in starter cultures used for food production, through which they enter the food chain and humans. In this way, they come into close contact with human microbiota [2,3].

A group of bacteria used for the production of vinegar, kefir, certain types of beer, nanocellulose, kombucha tea, nata de coco, and others, are acetic acid bacteria in the family *Acetobacteraceae* [4,5]. Two of the predominant genera in these processes are *Komagataeibacter* and *Acetobacter*. They are used for production at the industrial level and household needs. The natural environment of these bacteria are fruits and flowers, where different sugars and acids are available as carbon sources for their growth and multiplication. Circulation in the environment may enable the acetic acid bacteria to gain AMR via different gene exchange mechanisms from environmental microbiota. From the natural environment, such as vineyards, orchards, and meadows, the acetic acid bacteria are brought to the industrial and home environments. In this way, the acetic acid bacteria that originate from the surface of apples are reaching bioprocesses for the initiation of ethanol oxidation during apple cider vinegar production [6]. So far, researchers have mainly analyzed the biotechnologically relevant characteristics of acetic acid bacteria, such as the acetic acid production rate, tolerance to acetic acid and ethanol, and tolerance to high temperatures [7], but very rarely their AMR.

Data on the AMR of acetic acid bacteria have been mentioned in papers describing certain species of the *Acetobacter* genus in association with human diseases. In 2007, an isolate identified as *Acetobacter cibinongensis* was obtained from a blood sample of a 40 year old man in France suffering from bronchitis [8]. The antimicrobial drug susceptibility pattern could not be validated owing to the lack of assay reproducibility. However, the patient responded to the broad-spectrum antimicrobial therapy. In 2016, an isolate from a catheter of a child in Germany suffering from bacteremia was described as *Acetobacter indonesiensis* and was multidrug-resistant, with inhibition zones in an agar diffusion test only for imipenem, meropenem, fosfomycin, and tigecycline [9]. The isolate from the tracheostomy suction fluid of a 51 year old woman after lung transplantation was highly drug-resistant. This was *A. indonesiensis* as well, but showed in vitro activity against aminoglycosides, tetracyclines, imipenem, and ceftriaxone [10]. Recently, Wu et al. [11] identified, in a metagenomic approach, a genus *Acetobacter* in human fecal samples as one of the main genera carrying the top 20 antibiotic resistance gene types. So far, there are no data on the AMR of *Komagataeibacter* species.

To get more information on the AMR among two well-known acetic acid bacteria genera, we analyzed the resistance to selected antibiotics on a model group of different *Acetobacter* and *Komagataeibacter* species originating mainly from vinegars. To deepen this information to the DNA level, we searched in the genome sequences of type species for potential genetic information of AMR.

## 2. Materials and Methods

### 2.1. Strains of Acetic Acid Bacteria

The list of acetic acid bacteria used in this study is presented in Table 1. The model group presents 39 taxonomically well-characterized strains from the genera *Acetobacter* and *Komagataeibacter*. Although *Gluconacetobacter entanii* taxonomically belongs to the genus *Komagataeibacter*, this species remained in the genus *Gluconacetobacter* due to the absence of a cultivable form of the type strain of *Gluconacetobacter entanii* from any of the international culture collections of microorganisms.

Most of the strains originated from vinegars and were produced in different countries. The novel isolates of acetic acid bacteria were identified at the species level by the well-established method of 16S-23S rRNA ITS gene sequence analysis and the comparison to reference strains, as described before [12,13]. The sequences were deposited into the GenBank database under the accession numbers MZ747098, MZ747100, MZ747099, MZ725321, OL703587, MZ725320, OL703586, MZ725322, OL703585, OL703589, MZ725319, OL703588, MZ735454, and MT423517 for the strains *Acetobacter estunensis* AV380, *Acetobacter estunensis* AV390, *Acetobacter pasteurianus* AV366, *Acetobacter pasteurianus* SI3123, *Acetobacter pomorum* AV440, *Komagataeibacter melomenusus* SI3083, *Komagateibacter oboediens* AV371, *Komagataeibacter oboediens* SI3053, *Komagataeibacter pomaceti* AV445, *Komagataeibacter pomaceti* AV446, *Komagataeibacter pomaceti* SI3133, *Komagataeibacter saccharivorans* AV378, *Gluconacetobacter entanii* SI2035, and *Gluconacetobacter entanii* AV429, respectively.

The strains were revitalized from −80 °C on RAE medium composed of glucose (40 g/L), peptone (10 g/L), yeast extract (10 g/L), citric acid (1.37 g/L), Na_2_HPO_4_ × 2H_2_O (3.38 g/L), 1 vol% of ethanol, 1 vol% of acetic acid, and agar (10 g/L) [14]. The plates were incubated at 92–96% relative air humidity for 3 days at 30 °C. Additionally, strains were grown on media with mannitol (MA) and glucose (GY) at 30 °C for 3 days. The MA medium contained D-mannitol (25 g/L), peptone (3 g/L), yeast extract (5 g/L), and agar (15 g/L) (medium number 13 from the Belgian Coordinated Collections of Microorganisms), while the GY medium contained D-glucose (50 g/L), yeast extract (5 g/L), and agar (15 g/L) (modified medium 404 from the Belgian Coordinated Collections of Microorganisms).

### 2.2. Analysis of Antimicrobial Resistance (AMR)

The disk diffusion method adapted from EUCAST guidelines was used [15]. After successful revitalization, the strains were precultured onto RAE, GY, and MA media and incubated for three days at 30 °C; plates with the RAE medium were incubated at high relative air humidity. Then, the biomass from each plate was harvested into saline (0.85% NaCl) and the turbidity was adjusted to the value of the McFarland standard of 0.5. The bacterial suspension thus prepared was spread evenly with a sterile cotton swab over the entire surface of each medium. The antibiotic disks were applied onto plates. The following commercial antibiotic disks (BioRad, Hercules, USA) were applied onto the media: GMN10 for 10 μg of gentamicin, AMP10 for 10 μg of ampicillin, CHL30 for 30 μg of chloramphenicol, CIP5 for 5 μg of ciprofloxacin, ERY15 for 15 μg for erythromycin, and TMP5 for 5 μg of trimethoprim. Antibiotic resistance was compared among the strains by measuring the diameter of the growth inhibition zone around disks after two days of incubation at 30 °C of the inoculated medium, and in the case of the RAE medium, at a high relative air humidity.

### 2.3. Bioinformatics

The presence of homologues associated with AMR in the genome sequences of the type strains of acetic acid bacteria listed in Table 1 was analyzed using the online tool Resistance Gene Identifier (RGI) from the Comprehensive Antibiotic Resistance Database (CARD) [16]. The genome sequences of the type strains were downloaded from the NCBI database, annotated by RGI, and compared to the AMR reference database in CARD. Since none of the genome sequences had a homologue with a perfect matching, the selection criteria were extended to loose hits. The AMR homologues matching in length to the reference hit from 95% to 105% were selected as putative homologues. Additionally, a minimum of 25% amino acid identity matching the reference hit was set as a second criterion.

## 3. Results and Discussion

Acetic acid bacteria are widespread microorganisms in nature. They have been used to produce different foods and beverages [13] and pharmaceutical and medical products [17]. They are generally recognized as safe bacteria, but antibiotic resistance has not been systematically investigated in acetic acid bacteria.

In this study we analyzed the resistance of taxonomically well-defined representatives of *Acetobacter* and *Komagataeibacter* species, originating from different geographic areas, against antibiotics of different chemical structures and mechanisms of action. We selected ampicillin as a representative of bacterial cell wall synthesis inhibitor, chloramphenicol, erythromycin, and gentamicin as representative of bacterial protein synthesis inhibitors, and ciprofloxacin and trimethoprim as representative of bacterial DNA synthesis inhibitors. All these antibiotics belong to different antimicrobial classes: ampicillin to penicillins, chloramphenicol to phenicols, erythromycin to macrolides, gentamicin to aminoglycosides, ciprofloxacin to fluoroquinolones, and trimethoprim to diaminopyrimidines.

Since there is no standard method and medium defined yet for testing the antibiotic resistance of acetic acid bacteria, we used three different media which enable the growth of acetic acid bacteria. The main differences among media were in major carbon sources and pH values. The RAE growth medium has glucose and ethanol as carbon sources and a pH value of 3.5, the MA growth medium has mannitol and a pH of 6.7, and the GY medium has glucose and a pH of 6.9. The area of inhibition for each antibiotic differed among the media used (Tables S1–S3). Different factors can explain differences in the growth inhibition zones on different media against the same antibiotic. For example, in the case of trimethoprim, lower pH in the medium causes the ionization of trimethoprim, affecting its ability to cross the cell membrane in *E. coli* [18]. It is also possible that the medium components, such as sugars and acids, may direct the cells to different metabolic pathways, resulting in metabolites that may interact with antibiotics [19]. Then, the cell components, of which the synthesis has been inhibited by antibiotics, may be replaced by a compound present in the medium. Therefore, the formulation of an optimal medium that effectively supports bacterial growth but does not adversely affect its mechanism of action is difficult, especially for bacteria with specific growth requirements, including acetic acid bacteria.

**Figure 1:**
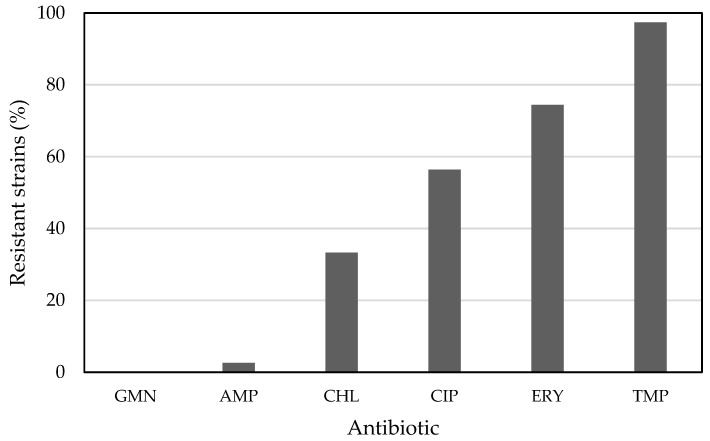
Acetic acid bacteria-resistant strains to gentamicin (GMN), ampicillin (AMP), chloramphenicol (CHL), ciprofloxacin (CIP), erythromycin (ERY), and trimethoprim (TMP).

Many acetic acid bacteria strains did not produce growth inhibition zones around the antibiotic disks (Tables S1–S3). These data were combined in one table (Table 2) according to the criterion that the absence of an inhibition zone on all of the applied growth media represents a resistant strain to a particular antibiotic. In this way, 97.4%, 74.4%, 56.4%, and 33.3% were resistant to trimethoprim, erythromycin, ciprofloxacin, and chloramphenicol, respectively (Figure 1). Moreover, 53.8% of all strains were resistant to three or more antimicrobial classes [20], and thus represented multidrug-resistant strains. The most common profile of multiple resistance was trimethoprim/ciprofloxacin/erythromycin. Only a few strains were resistant to ampicillin or gentamicin; on all three media, only one strain was resistant towards ampicillin, and none against gentamicin (Figure 1). Separate figures for *Acetobacter* and *Komagataeibacter* species show that a substantial difference in resistance against chloramphenicol, ciprofloxacin, and erythromycin exists (Figure S1); however, this has to be proved with a higher number of tested strains from each genus in the future.

Of all tested strains and growth media, only the strain *Komagataeibacter melaceti* AV382^T^ cultured on the RAE medium was sensitive to trimethoprim. Its growth inhibition zone (8–9 mm) was obvious and repeatedly detected (Table S1). Resistance to trimethoprim is caused by decreased cell permeability and modifications in the bacterial target for trimethoprim, i.e., dihydrofolate reductase [21]. Trimethoprim resistance can also originate from cross-resistance to other antibiotics [22]. Since most strains resistant to trimethoprim in this work also possess resistance to some other antibiotics (i.e., erythromycin, ciprofloxacin, and chloramphenicol), a general nonspecific mechanism, such as transport out of the cell, may be responsible for trimethoprim resistance. Although trimethoprim resistance has been detected in almost all strains, a trimethoprim-sensitive strain (Table 2) suggests that trimethoprim resistance is not an intrinsic characteristic for acetic acid bacteria.

Erythromycin resistance has been well-studied in another group of food-grade bacteria, lactic acid bacteria (LAB). A total of 97 out of 155 isolates of LAB from human feces were resistant to erythromycin, and 19 of them carried *ermB*, coding for erythromycin esterase. The gene was also successfully transferable to *Enterococcus faecalis* [23]. Bacteria have also developed other mechanisms to circumvent the action of macrolides, such as decreasing intracellular concentration via the use of efflux pumps, ribosome modification, ribosome protection, and macrolide phosphotransferase-mediated modification [23]. In the presented study, none of the strains of *Acetobacter* and *Komagataeibacter* resisted erythromycin on the RAE medium (Table S1), in contrast to the successful growth of some strains in the presence of erythromycin on the GY (Table S2) and MA media (Table S3). This suggests the instability of erythromycin at a low pH in the RAE medium; an observation also supported by the study of Brisaert, who showed that the stability of erythromycin was negatively affected by a low pH [24].

Ciprofloxacin is an ampholytic compound with a pK_a_ value of 6.09 for the carboxylic group and 8.74 for the nitrogen on the piperazinyl ring [25]. Zwitterion structure formed at a pH of 7.4 and was susceptible to photodegradation. Maximal stability of ciprofloxacin was observed at a pH of 3.0 and 4.0 [26]. This may also explain why most of the analyzed strains of *Acetobacter* and *Komagataeibacter* were resistant against this drug in the RAE medium, of which the pH value was 3.5. Interestingly, the only exception to this was the strain *Gluconacetobacter entanii* AV429. A total of 66.7% and 64.1% of strains on the GY and MA media were ciprofloxacin-resistant. Ciprofloxacin targets bacterial topoisomerase II (DNA gyrase), which are involved in DNA replication and transcription [27]. Generally, the mutation in genes coding for gyrase (*gyrA*) is responsible for the resistant phenotype; however, other mechanisms, especially efflux pumps, may also prevent the intracellular action of ciprofloxacin [28].

Chloramphenicol sensitivity among strains of acetic acid bacteria has been observed on all three media, and also on the RAE medium with an acidic pH (Table S1), which is probably the result of chloramphenicol pH stability over a wide pH range [29]. However, only 13 strains, distributed among the *Acetobacter* and *Komagataeibacter* genera, resisted chloramphenicol on all three media (Table 2), suggesting that other factors may be involved in the susceptibility to this drug. Bacteria have developed different chloramphenicol resistance mechanisms, a very specific enzymatic inactivation of the drug by chloramphenicol acetyltransferase, but also efflux drug removal, ribosome protection, and the nitro reduction of chloramphenicol [30].

Ampicillin resistance was detected on each of the three media; however, only one strain exhibited resistance on all three media. The number of strains resistant to ampicillin on the RAE and GY media was the same, but not the same strains exhibited resistance on both media. The number of strains resistant to ampicillin was the lowest on the MA medium. The previous study showed the instability of ampicillin at a higher pH [31]; however, a high number of ampicillin resistance strains on the GY medium (pH 6.9) (Table S2) in contrast to the RAE medium (pH 3.5) (Table S1) suggests that other factors are involved in the ampicillin resistance of acetic acid bacteria on these media.

We detected a high difference in the number of strains resistant to gentamicin among the culture media. The number of strains resisting gentamicin on the GY and MA media was one and three, respectively, but on the RAE medium it was 22. Since gentamicin is stable at a pH of 2–14, the reason for these differences has to be in other factors. Gentamicin resistance is often associated with aminoglycoside-modifying enzymes such as acetyltransferases, phosphotransferases, and methyltransferases. Besides, target site modification and drug efflux may also be responsible for gentamicin resistance [32].

The antibiotic resistome prediction in the genome sequences of type strains revealed matching regions to known molecular determinants of antibiotic resistance (Table S4). These genetic determinants corresponded to five resistance mechanisms: antibiotic efflux, antibiotic target alteration, antibiotic inactivation, reduced permeability to the antibiotic, and antibiotic target replacement (Table 3). The proportion of individual mechanisms was similar in each genus (Table S5). The highest number of homologues, among them AcrA, MdtA, MexB, MuxB, OpmB, OprM, AmvA, BaeR, RanA, EmrE, QacL, TriC, MdsC, MexK, RosA, EmrB, FarA, MexL, RosB, TxR, SdiA, KdpE, MsbA, QacE, MexS, MtrA, and NmcR, were in the category antibiotic efflux (Table S4). These putative pumps may transport different antibiotics out of the cell. Further on, at least one homologue putatively encoding β-lactamase was detected for each of the analyzed reference strains. Since this is the most well-known mechanism for ampicillin deactivation [33], ampicillin resistance was expected for these strains and confirmed for each reference strain on at least one of the three growth media. In two reference strains, a genetic determinant putatively coding for chloramphenicol acetyltransferase was detected; both strains also exhibited resistance to chloramphenicol on at least one of the growth media. The correlation between the resistance of acetic acid bacteria to the specific antibiotic and genetic determinants responsible for it needs to be further elucidated by genetic studies in acetic acid bacteria.

## 4. Conclusions

This work is the first pilot systematic study on AMR in the genera *Acetobacter* and *Komagataeibacter*. Although the assessment of AMR among acetic acid bacteria is confounded due to the lack of standards for testing, these results have undoubtedly revealed widespread AMR among *Acetobacter* and *Komagataeibacter* species. This is just a first step in this research field that needs further studies to identify the exact genetic determinants and molecular mechanisms for AMR in acetic acid bacteria, but also to find out if this genetic information is transferable to human pathogens and to environmental bacteria, from where it can further spread to clinically relevant bacteria. It is also crucial that, in the future, a standardized method is established for the analysis of antibiotic resistance in this group of bacteria, or separately for each genus of acetic acid bacteria.

## Figures and Tables

**Table 1:** List of strains used in this study.

Species and Strain Designation	Source of Isolation
*Acetobacter aceti* DSM 3508^T^	Beechwood shavings of vinegar plant
*Acetobacter estunensis* AV380	Apple cider vinegar (company Apis Vita, Fram, Slovenia)
*Acetobacter estunensis* AV390	Apple cider vinegar (company Apis Vita, Fram, Slovenia)
*Acetobacter orleanensis* IFO 13752^T^	Beer (Belgium)
*Acetobacter pasteurianus* DSM 3509^T^	Beer (The Netherlands)
*Acetobacter pasteurianus* AV366	Apple cider vinegar (company Apis Vita, Fram, Slovenia)
*Acetobacter pasteurianus* JK_T6K1	Apple cider vinegar (company Šampionka Renče, Volčja Draga, Slovenia)
*Acetobacter pasteurianus* JK_T1K1	Apple cider vinegar (company Šampionka Renče, Volčja Draga, Slovenia)
*Acetobacter pasteurianus* BJK_1B	Apple cider vinegar (company Šampionka Renče, Volčja Draga, Slovenia)
*Acetobacter pasteurianus* SI3123	Apple cider vinegar (company Kisarna Simonič, Zgornja Ščavnica, Slovenia)
*Acetobacter pomorum* LMG 18848^T^	Apple cider vinegar (Germany)
*Acetobacter pomorum* AV440	Apple cider vinegar (company Apis Vita, Fram, Slovenia)
*Acetobacter tropicalis* IFO 16470^T^	Coconut (Indonesia)
*Komagataeibacter europaeus* LMG 18494	Red wine vinegar produced in submerged bioreactor (company Kolinska, Ljubljana, Slovenia)
*Komagataeibacter europaeus* LMG 20956	Apple cider vinegar (company Kolinska, Ljubljana, Slovenia)
*Komagataeibacter hansenii* DSM 5602^T^	Vinegar (Israel)
*Komagataeibacter hansenii* LMG 23726	Kombucha (India)
*Komagataeibacter kakiaceti* LMG 26206^T^	Kaki vinegar (Japan)
*Komagataeibacter maltaceti* LMG 1529^T^	Malt vinegar brewery acetifiers
*Komagataeibacter maltaceti* SKU 1109	Fruit (Thailand)
*Komagataeibacter medellinensis* LMG 1693^T^	Vinegar
*Komagataeibacter melaceti* AV382^T^	Apple cider vinegar (company Apis Vita, Fram, Slovenia)
*Komagataeibacter melomenusus* AV436^T^	Apple cider vinegar (company Apis Vita, Fram, Slovenia)
*Komagataeibacter melomenusus* SI3083	Apple cider vinegar (company Kisarna Simonič, Zgornja Ščavnica, Slovenia)
*Komagataeibacter nataicola* LMG 1536^T^	Nata de coco (Philippines)
*Komagateibacter oboediens* AV371	Apple cider vinegar (company Apis Vita, Fram, Slovenia)
*Komagataeibacter oboediens* BJK_8C	Apple cider vinegar (company Šampionka Renče, Volčja Draga, Slovenia)
*Komagataeibacter oboediens* SI3053	Apple cider vinegar (company Kisarna Simonič, Zgornja Ščavnica, Slovenia)
*Komagataeibacter pomaceti* T5K1^T^	Apple cider vinegar (company Šampionka Renče, Volčja Draga, Slovenia)
*Komagataeibacter pomaceti* AV445	Apple cider vinegar (company Apis Vita, Fram, Slovenia)
*Komagataeibacter pomaceti* AV446	Apple cider vinegar (company Apis Vita, Fram, Slovenia)
*Komagataeibacter pomaceti* SI3133	Apple cider vinegar (company Kisarna Simonič, Zgornja Ščavnica, Slovenia)
*Komagataeibacter rhaeticus* DSM 16663^T^	Organic apple juice (Italy)
*Komagataeibacter saccharivorans* LMG 1582^T^	Beet juice (Germany)
*Komagataeibacter saccharivorans* AV378	Apple cider vinegar (company Apis Vita, Fram, Slovenia)
*Komagataeibacter saccharivorans* JK_3A	Apple cider vinegar (company Šampionka Renče, Volčja Draga, Slovenia)
*Komagataeibacter swingsii* LMG 22125^T^	Organic apple juice (Italy)
*Gluconacetobacter entanii* SI2035	Apple cider vinegar (company Kisarna Simonič, Zgornja Ščavnica, Slovenia)
*Gluconacetobacter entanii* AV429	Apple cider vinegar (company Apis Vita, Fram, Slovenia)

**Table 2:** Resistance of *Acetobacter* and *Komagataeibacter* strains against antibiotics: gentamicin (GMN), ampicillin (AMP), chloramphenicol (CHL), ciprofloxacin (CIP), erythromycin (ERY) and trimethoprim (TMP). The X-label represents the growth arrest of a particular strain around the antibiotic disk on all three tested growth media.

Species and Strain Designation	GMN	AMP	CHL	CIP	ERY	TMP
*Acetobacter aceti* DSM 3508^T^				X	X	X
*Acetobacter estunensis* AV380			X	X	X	X
*Acetobacter estunensis* AV390			X	X	X	X
*Acetobacter orleanensis* IFO 13752^T^				X	X	X
*Acetobacter pasteurianus* DSM 3509^T^				X		X
*Acetobacter pasteurianus* AV366			X			X
*Acetobacter pasteurianus* JK_T6K1			X	X	X	X
*Acetobacter pasteurianus* JK_T1K1			X	X	X	X
*Acetobacter pasteurianus* BJK_1B			X	X		X
*Acetobacter pasteurianus* SI3123			X	X		X
*Acetobacter pomorum* LMG 18848^T^			X	X		X
*Acetobacter pomorum* AV440			X	X		X
*Acetobacter tropicalis* IFO 16470^T^					X	X
*Komagataeibacter europaeus* LMG 18494					X	X
*Komagataeibacter europaeus* LMG 20956					X	X
*Komagataeibacter hansenii* DSM 5602^T^					X	X
*Komagataeibacter hansenii* LMG 23726					X	X
*Komagataeibacter kakiaceti* LMG 26206^T^			X	X	X	X
*Komagataeibacter maltaceti* LMG 1529^T^					X	X
*Komagataeibacter maltaceti* SKU 1109					X	X
*Komagataeibacter medellinensis* LMG 1693^T^				X	X	X
*Komagataeibacter melaceti* AV382^T^			X			
*Komagataeibacter melomenusus* AV436^T^						X
*Komagataeibacter melomenusus* SI3083				X	X	X
*Komagataeibacter nataicola* LMG 1536^T^				X		X
*Komagateibacter oboediens* AV371			X	X	X	X
*Komagataeibacter oboediens* BJK_8C				X	X	X
*Komagataeibacter oboediens* SI3053					X	X
*Komagataeibacter pomaceti* T5K1^T^					X	X
*Komagataeibacter pomaceti* AV445					X	X
*Komagataeibacter pomaceti* AV446					X	X
*Komagataeibacter pomaceti* SI3133		X		X	X	X
*Komagataeibacter rhaeticus* DSM 16663^T^			X		X	X
*Komagataeibacter saccharivorans* LMG 1582^T^				X	X	X
*Komagataeibacter saccharivorans* AV378				X	X	X
*Komagataeibacter saccharivorans* JK_3A				X	X	X
*Komagataeibacter swingsii* LMG 22125^T^					X	X
*Gluconacetobacter entanii* SI2035				X	X	X
*Gluconacetobacter entanii* AV429						X

**Table 3:** Resistance mechanisms of putative antimicrobial resistance gene families identified in type strains of *Acetobacter* and *Komagataeibacter* species listed in Table 1.

Resistance Mechanism	AMR Gene Family	Number of Homologues
Antibiotic efflux	Major facilitator superfamily (MFS) antibiotic efflux pump, resistance-nodulation-cell division (RND) antibiotic efflux pump, multidrug and toxic compound extrusion (MATE) transporter, ATP-binding cassette (ABC) antibiotic efflux pump, small multidrug resistance (SMR) antibiotic efflux pump.	648
Antibiotic target alteration	Pmr phosphoethanolamine transferase, antibiotic-resistant murA transferase, rifampin glycosyltransferase, streptogramin vat acetyltransferase, MCR phosphoethanolamine transferase, fosfomycin thiol transferase, fluoroquinolone-resistant gyrB, daptomycin-resistant cls.	259
Antibiotic inactivation	LHK beta-lactamase, AmpC-type beta-lactamase, NmcA beta-lactamase, subclass B3 LRA beta-lactamase, AIM beta-lactamase, CRD3 beta-lactamase, DHT2 beta-lactamase, SRT beta-lactamase, CGA beta-lactamase, SST beta-lactamase, chloramphenicol phosphotransferase, chloramphenicol acetyltransferase, tetracycline inactivation enzyme, fosfomycin thiol transferase.	62
Reduced permeability to antibiotic	General bacterial porin with reduced permeability to beta-lactams, intrinsic peptide antibiotic resistant Lps, outer membrane porin (Opr).	27
Antibiotic target replacement	Methicillin-resistant PBP2.	2

## Data Availability

Not applicable.

## Supplementary Materials

The following are available online at https://www.mdpi.com/article/10.3390/ijerph19010463/s1, Table S1: Antibiogram of *Acetobacter* and *Komagataeibacter* species on RAE medium. No inhibition zone is presented as /; Table S2: Antibiogram of *Acetobacter* and *Komagataeibacter* species on GY medium. No inhibition zone is presented as /; Table S3: Antibiogram of *Acetobacter* and *Komagataeibacter* species on MA medium. No inhibition zone is presented as /; Table S4: List of homologues to antibiotic resistance genes in the genome sequences of the type strains analyzed in this study; Table S5: Resistance mechanisms of putative resistance gene families identified in type strains of *Acetobacter* species (A) and *Komagataeibacter* species (B). Figure S1: Strains of *Acetobacter* species (A) and *Komagataeibacter* species (B) resistant to gentamicin (GMN), ampicillin (AMP), chloramphenicol (CHL), ciprofloxacin (CIP), erythromycin (ERY), and trimethoprim (TMP).
