# Supplementary material for: Antimicrobial Resistance of Acetobacter and Komagataeibacter Species Originating from Vinegars
Source: Int J Environ Res Public Health. 2022 Jan 1;19(1):463. doi: 10.3390/ijerph19010463 (PMC8744987; doi:10.3390/ijerph19010463)
Supplement: Supplementary file 1 [file ijerph-19-00463-s001.zip › Figure S1.pptx]

## Slide 1
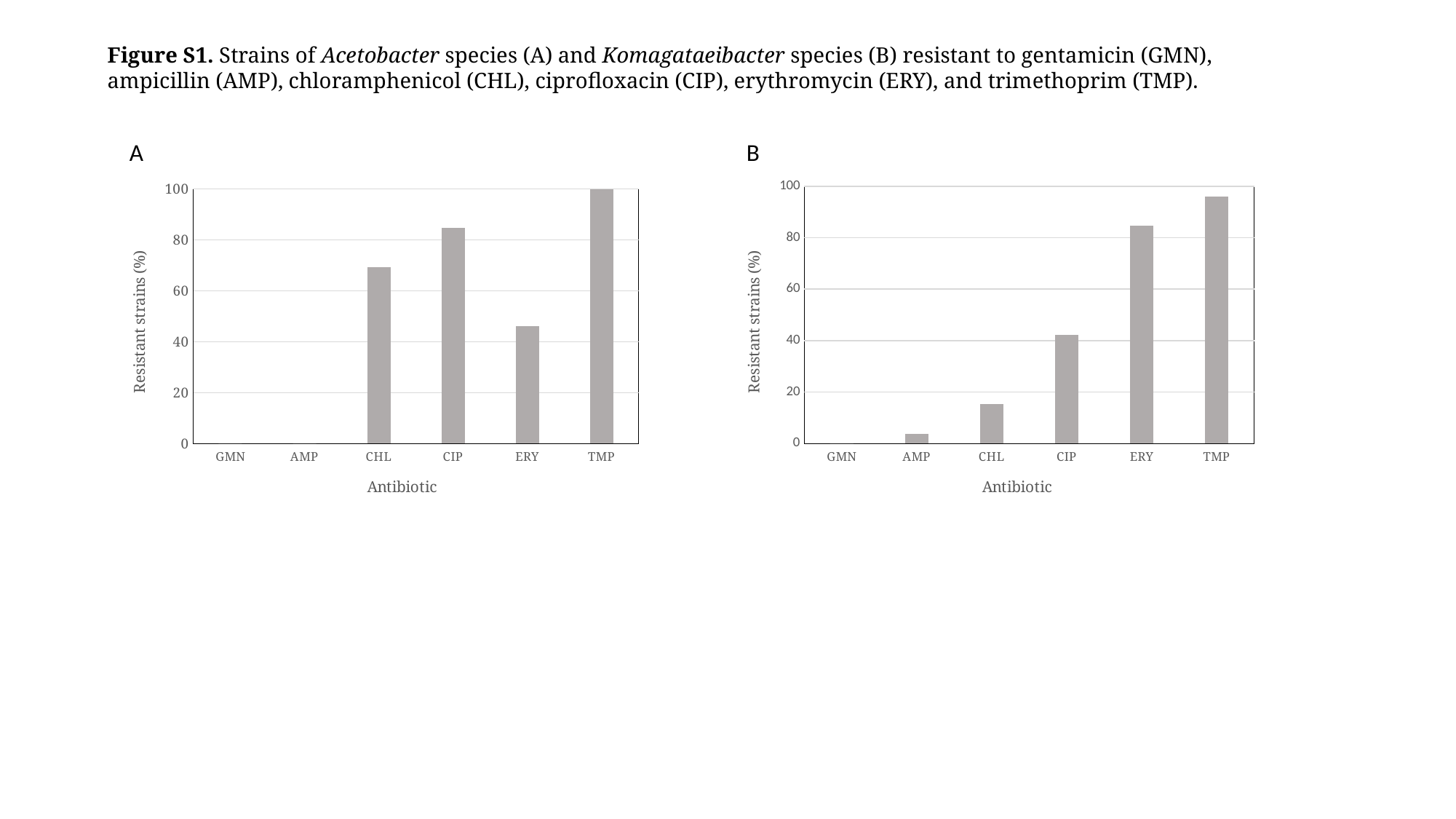

Figure S1. Strains of Acetobacter species (A) and Komagataeibacter species (B) resistant to gentamicin (GMN), ampicillin (AMP), chloramphenicol (CHL), ciprofloxacin (CIP), erythromycin (ERY), and trimethoprim (TMP).
A
B
### Chart
| Category | Resistant strains of Acetobacter |
|---|---|
| GMN | 0.0 |
| AMP | 0.0 |
| CHL | 69.2 |
| CIP | 84.6 |
| ERY | 46.1 |
| TMP | 100.0 |
### Chart
| Category | |
|---|---|
| GMN | 0.0 |
| AMP | 3.8 |
| CHL | 15.4 |
| CIP | 42.3 |
| ERY | 84.6 |
| TMP | 96.1 |
