# Supplementary material for: Antimicrobial Resistance of Acetobacter and Komagataeibacter Species Originating from Vinegars
Source: Int J Environ Res Public Health. 2022 Jan 1;19(1):463. doi: 10.3390/ijerph19010463 (PMC8744987; doi:10.3390/ijerph19010463)
Supplement: Supplementary file 1 [file ijerph-19-00463-s001.zip › TableS1_antibiogram_RAE.pdf]

Table S1. Antibiogram of *Acetobacter* and *Komagataeibacter* species on RAE medium. No inhibition zone is presented as /.

Species and strain designation	Ampicillin	Chloramphenicol	Gentamicin	Trimethoprim	Ciprofloxacin	Erytromycin
Acetobacter aceti DSM 3508^T	11 mm	14 mm	9 mm	/	/	/
Acetobacter estunensis AV380	/	/	24 mm	/	/	/
Acetobacter estunensis AV390	21 mm	/	14 mm	/	/	/
Acetobacter orleanensis IFO 13752^T	/	/	/	/	/	/
Acetobacter pasteurianus DSM 3509^T	10 mm	/	8 mm	/	/	/
Acetobacter pasteurianus AV366	22 mm	/	10 mm	/	/	/
Acetobacter pasteurianus JK_T6K1	21 mm	/	8 mm	/	/	/
Acetobacter pasteurianus JK_T1K1	16 mm	/	10 mm	/	/	/
Acetobacter pasteurianus BJK_1B	23 mm	/	9 mm	/	/	/
Acetobacter pasteurianus SI3123	18 mm	/	20 mm	/	/	/
Acetobacter pomorum LMG 18848^T	13 mm	/	10 mm	/	/	/
Acetobacter pomorum AV440	10 mm	/	9 mm	/	/	/
Acetobacter tropicalis IFO 16470^T	/	13 mm	/	/	/	/
Komagataeibacter europaeus LMG 18494	14 mm	/	/	/	/	/
Komagataeibacter europaeus LMG 20956	20 mm	12 mm	8 mm	/	/	/
Komagataeibacter hansenii DSM 5602^T	/	12 mm	/	/	/	/
Komagataeibacter hansenii LMG 23726	/	15 mm	/	/	/	/
Komagataeibacter kakiaceti LMG 26206^T	15 mm	/	8 mm	/	/	/
Komagataeibacter maltaceti LMG 1529^T	/	/	/	/	/	/
Komagataeibacter maltaceti SKU 1109	/	/	/	/	/	/
Komagataeibacter medellinensis LMG 1693^T	10 mm	/	/	/	/	/
Komagataeibacter melaceti AV382^T	13 mm	/	9 mm	8 mm	/	/
Komagataeibacter melomenusus AV436^T	10 mm	/	/	/	/	/
Komagataeibacter melomenusus SI3083	11 mm	15 mm	/	/	/	/
Komagataeibacter nataicola LMG 1536^T	26 mm	/	19 mm	/	/	/
Komagateibacter oboediens AV371	15 mm	/	/	/	/	/
Komagataeibacter oboediens BJK_8C	17 mm	/	/	/	/	/
Komagataeibacter oboediens SI3053	16 mm	/	/	/	/	/
Komagataeibacter pomaceti T5K1^T	10 mm	15 mm	10 mm	/	/	/

Komagataeibacter pomaceti AV445	9 mm	/	/	/	/	/
Komagataeibacter pomaceti AV446	12 mm	/	/	/	/	/
Komagataeibacter pomaceti SI3133	/	/	/	/	/	/
Komagataeibacter rhaeticus DSM 16663^T	/	/	/	/	/	/
Komagataeibacter saccharivorans LMG 1582^T	22 mm	/	8 mm	/	/	/
Komagataeibacter saccharivorans AV378	22 mm	/	/	/	/	/
Komagataeibacter saccharivorans JK_3A	12 mm	/	/	/	/	/
Komagataeibacter swingsii LMG 22125^T	18 mm	/	/	/	/	/
Gluconacetobacter entanii SI2035	/	/	/	/	/	/
Gluconacetobacter entanii AV429	/	/	/	/	20 mm	/
