# Supplementary material for: Antimicrobial Resistance of Acetobacter and Komagataeibacter Species Originating from Vinegars
Source: Int J Environ Res Public Health. 2022 Jan 1;19(1):463. doi: 10.3390/ijerph19010463 (PMC8744987; doi:10.3390/ijerph19010463)
Supplement: Supplementary file 1 [file ijerph-19-00463-s001.zip › TableS2_antibiogram_GY.pdf]

Table S2. Antibiogram of *Acetobacter* and *Komagataeibacter* species on GY medium. No inhibition zone is presented as /.

**Species and strain designation**	**Ampicillin**	**Chloramphenicol**	**Gentamicin**	**Trimethoprim**	**Ciprofloxacin**	**Erytromycin**
Acetobacter aceti DSM 3508^{T}	/	/	12 mm	/	/	/
Acetobacter estunensis AV380	/	/	14 mm	/	/	/
Acetobacter estunensis AV390	8 mm	/	12 mm	/	/	/
Acetobacter orleanensis IFO 13752^{T}	/	/	10 mm	/	/	/
Acetobacter pasteurianus DSM 3509^{T}	14 mm	/	11 mm	/	/	30 mm
Acetobacter pasteurianus AV366	31 mm	/	19 mm	/	16 mm	20 mm
Acetobacter pasteurianus JK_T6K1	32 mm	/	11 mm	/	/	/
Acetobacter pasteurianus JK_T1K1	24 mm	/	13 mm	/	/	/
Acetobacter pasteurianus BJK_1B	28 mm	/	11 mm	/	/	23 mm
Acetobacter pasteurianus SI3123	18 mm	/	12 mm	/	/	/
Acetobacter pomorum LMG 18848^{T}	28 mm	11 mm	13 mm	/	/	/
Acetobacter pomorum AV440	16 mm	/	11 mm	/	/	30 mm
Acetobacter tropicalis IFO 16470^{T}	/	/	/	/	/	/
Komagataeibacter europaeus LMG 18494	36 mm	/	11 mm	/	13 mm	/
Komagataeibacter europaeus LMG 20956	/	/	/	/	/	/
Komagataeibacter hansenii DSM 5602^{T}	/	/	10 mm	/	12 mm	/
Komagataeibacter hansenii LMG 23726	/	11 mm	11 mm	/	14 mm	/
Komagataeibacter kakiaceti LMG 26206^{T}	11 mm	/	13 mm	/	/	/
Komagataeibacter maltaceti LMG 1529^{T}	16 mm	/	15 mm	/	15 mm	/
Komagataeibacter maltaceti SKU 1109	32 mm	/	11 mm	/	19 mm	/
Komagataeibacter medellinensis LMG 1693^{T}	16 mm	14 mm	8 mm	/	/	/
Komagataeibacter melaceti AV382^{T}	/	/	12 mm	/	/	/
Komagataeibacter melomenusus AV436^{T}	44 mm	/	13 mm	/	28 mm	47 mm
Komagataeibacter melomenusus SI3083	20 mm	21 mm	10 mm	/	/	/
Komagataeibacter nataicola LMG 1536^{T}	36 mm	19 mm	13 mm	/	/	11 mm
Komagateibacter oboediens AV371	18 mm	/	8 mm	/	/	/
Komagataeibacter oboediens BJK_8C	20 mm	/	8 mm	/	/	/
Komagataeibacter oboediens SI3053	26 mm	/	12 mm	/	/	/
Komagataeibacter pomaceti T5K1^{T}	10 mm	12 mm	10 mm	/	16 mm	/

Komagataeibacter pomaceti AV445	10 mm	23 mm	14 mm	/	14 mm	/
Komagataeibacter pomaceti AV446	/	20 mm	11 mm	/	16 mm	/
Komagataeibacter pomaceti SI3133	/	15 mm	12 mm	/	/	/
Komagataeibacter rhaeticus DSM 16663^{T}	29 mm	17 mm	14 mm	/	11 mm	/
Komagataeibacter saccharivorans LMG 1582^{T}	14 mm	/	9 mm	/	/	/
Komagataeibacter saccharivorans AV378	35 mm	/	/	/	/	/
Komagataeibacter saccharivorans JK_3A	14 mm	/	8 mm	/	/	/
Komagataeibacter swingsii LMG 22125^{T}	40 mm	35 mm	20 mm	/	28 mm	/
Gluconacetobacter entanii SI2035	/	/	11 mm	/	/	/
Gluconacetobacter entanii AV429	11 mm	13 mm	12 mm	/	35 mm	/
