# Supplementary material for: Antimicrobial Resistance of Acetobacter and Komagataeibacter Species Originating from Vinegars
Source: Int J Environ Res Public Health. 2022 Jan 1;19(1):463. doi: 10.3390/ijerph19010463 (PMC8744987; doi:10.3390/ijerph19010463)
Supplement: Supplementary file 1 [file ijerph-19-00463-s001.zip › TableS5.pptx]

## Slide 1
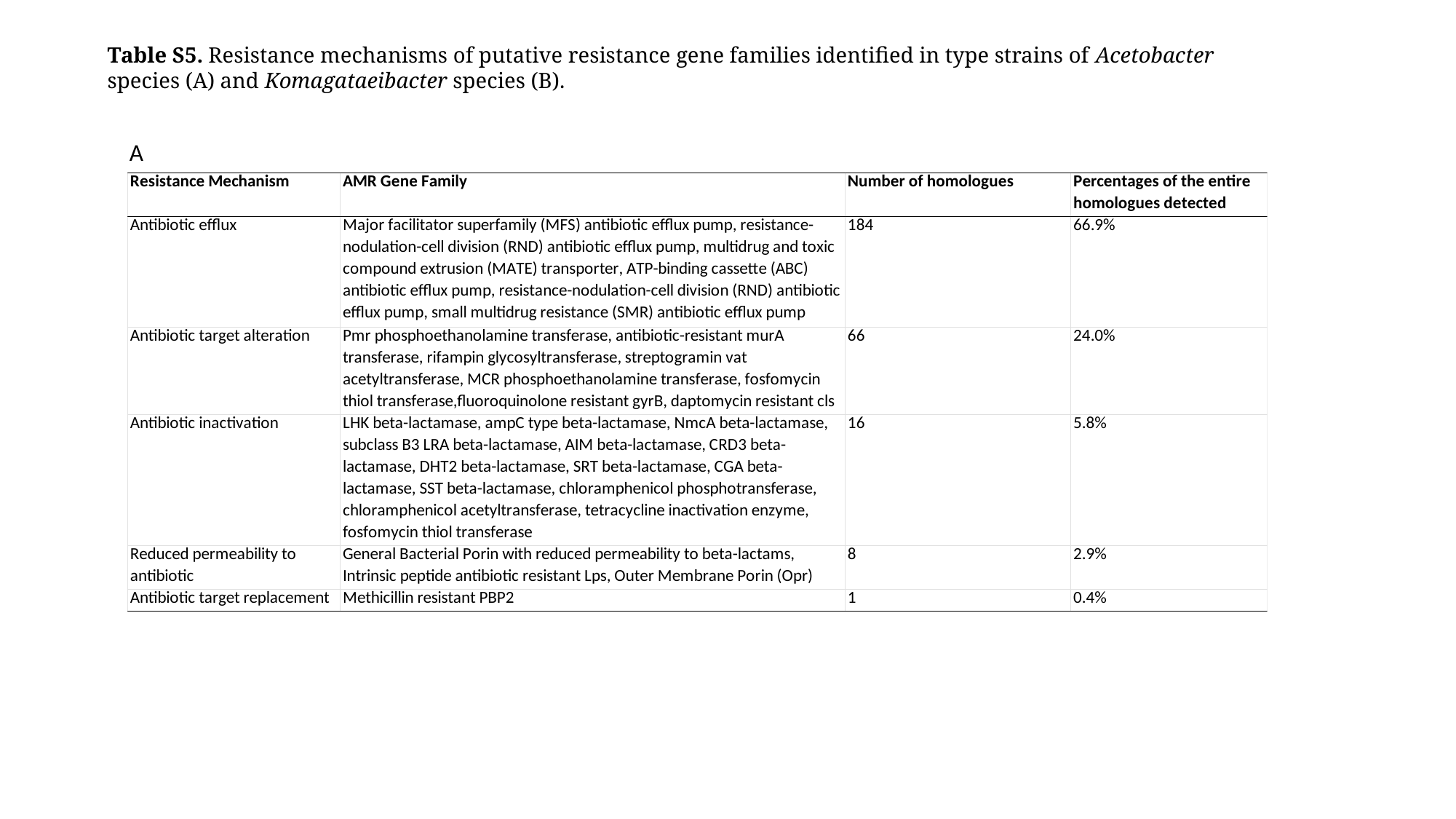

Table S5. Resistance mechanisms of putative resistance gene families identified in type strains of Acetobacter species (A) and Komagataeibacter species (B).
A

## Slide 2
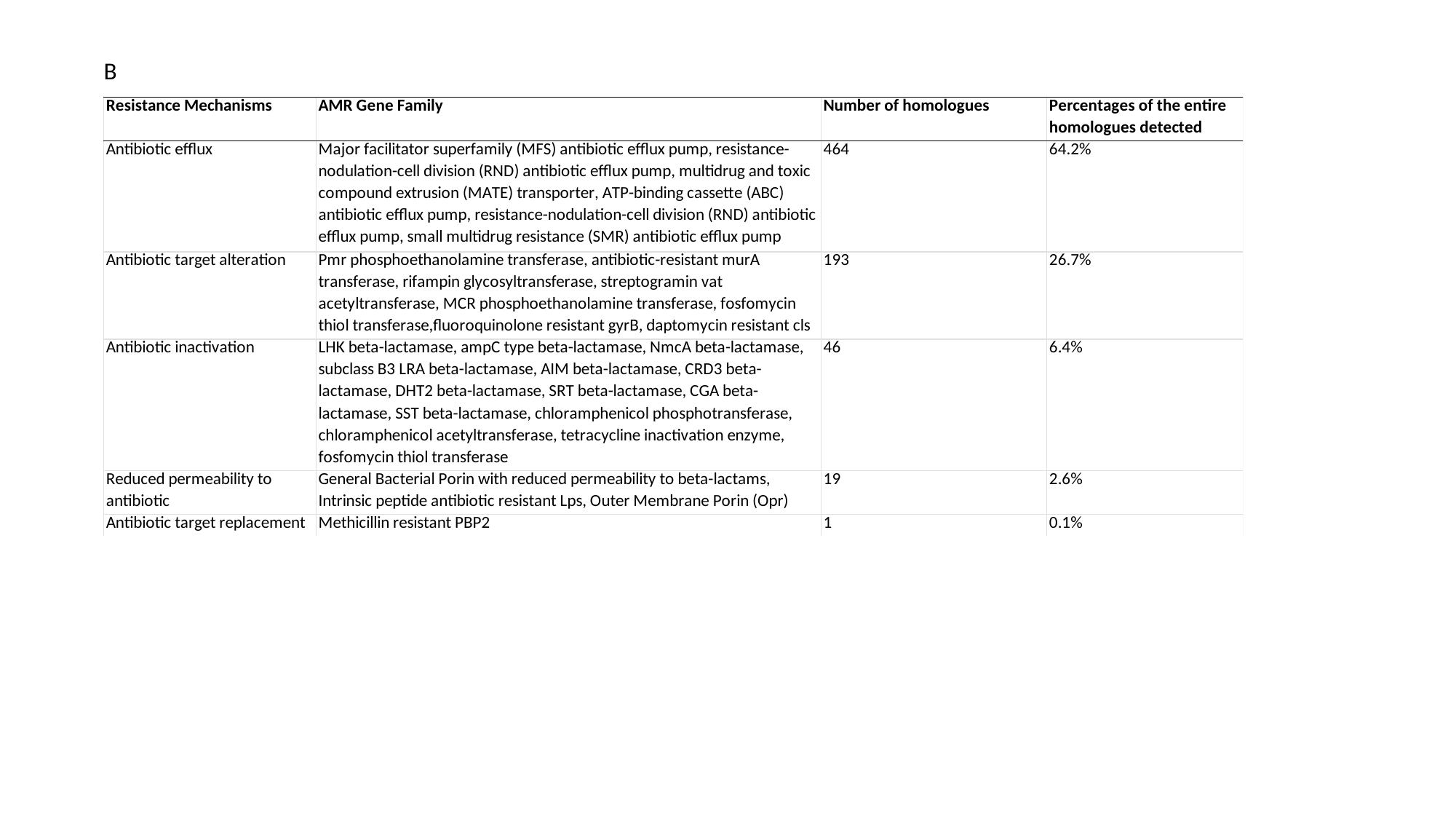

B
